# Nature versus nurture? Consequences of short captivity in early stages

**DOI:** 10.1002/ece3.3555

**Published:** 2017-12-01

**Authors:** Jose L. Horreo, America G. Valiente, Alba Ardura, Aida Blanco, Claudia Garcia‐Gonzalez, Eva Garcia‐Vazquez

**Affiliations:** ^1^ Department of Biodiversity and Evolutionary Biology National Museum of Natural Sciences (CSIC) Madrid Spain; ^2^ Department of Functional Biology University of Oviedo Oviedo Spain

**Keywords:** domestication, fitness, isotope, migration, population restoration, reintroduction, *Salmo salar*, zoo

## Abstract

Biological changes occurring as a consequence of domestication and/or captivity are not still deeply known. In Atlantic salmon (S*almo salar*), endangered (Southern Europe) populations are enhanced by supportive breeding, which involves only 6 months of captive rearing following artificial spawning of wild‐collected adults. In this work, we assess whether several fitness‐correlated life‐history traits (migratory behavior, straying rate, age at maturity, and growth) are affected by early exposure to the captive environment within a generation, before reproduction thus before genetic selection. Results showed significant differences in growth and migratory behavior (including straying), associated with this very short period of captivity in natural fish populations, changing even genetic variability (decreased in hatchery‐reared adults) and the native population structure within and between rivers of the species. These changes appeared within a single generation, suggesting very short time of captivity is enough for initiating changes normally attributed to domestication. These results may have potential implications for the long‐term population stability/viability of species subjected to restoration and enhancement processes and could be also considered for the management of zoo populations.

## 1 BACKGROUND

The genetic and physiological changes occurring as a consequence of domestication are not easy to generalize because they obviously depend on the particular species, rearing system, and domestication time. Most domestication processes involve behavioral, morphological, and physiological changes (e.g., faster growth), and generally a loss of genetic diversity in domesticated stocks (Horreo, 2015; Lacy, 1987). Increased genetic and phenotypic flexibilities in domesticates (as compared to wild counterparts) have been proposed to explain behaviors and their consequences that would be anomalous in the wild but are successful in domestic populations, including, for example, fertile offspring in crosses between related taxa (Dobney & Larson, 2006). Many genes of moderate effect would be involved in the differences between domesticated stocks and their wild‐type progenitors (Albert et al., 2011; Kukekova et al., 2011; Trut, Oskina, & Kharlamova, 2009), and different genomic imprinting (inheritable epigenetic change) in domestic and wild individuals would contribute to explain at least a part of the changes occurring in domestication processes (e.g., O'Doherty, MacHugh, Spillane, & Magee, 2015; Trut et al., 2009; Wilkins, Wrangham, & Fitch, 2014).

Whatever the causative mechanisms and genes involved, species that adapt to captivity experience genetic changes, and the number of generations in captivity is directly associated with the magnitude of genetic differences between wild types and domesticates (Frankham, 2008). Divergence of captive populations from wild populations is of great importance to conservation programs. In an attempt to save endangered populations, managers, conservationists, and scientists have developed strategies of supportive breeding, which can include artificial crosses in captivity, as well as release of offspring into the wild. However, these well‐intentioned initiatives can promote maladaptive traits as natural selection and can be distorted by human intervention (e.g., Bestgena, Zelaskoa, Comptona, & Chartb, 2008; Jónás et al., 2010; Levin, Zabel, & Williams, 2001; Massaro et al., 2013). Reducing the number of captivity generations is expected to reduce the extent of adaptation to captivity (Frankham, 2008) and potentially increase the success of reintroduction of populations into the wild from captivity. However, the maximum number of captivity generations that can occur without hindering future adaptation in the wild is not clear. Some authors postulate that changes from wild to domestic type can occur quite rapidly (Jensen, 2014; Trut et al., 2009), even within only one generation of domestication (Christie, Marine, French, & Blouin, 2011). This includes heritable changes in the expression of hundreds of genes, probably involved in adaptation to high densities in hatchery conditions compared to the wild environment (Christie, Marine, Fox, French, & Blouin, 2016). In summary, it appears that durable genetic changes in captive populations can occur in as little as one generation and that these changes can have effects across multiple generations (e.g., epigenetic and heritable changes; Jonsson & Jonsson, 2014).

One example of a recently domesticated species with co‐occurring wild populations is Atlantic salmon (*Salmo salar*): domestic stocks are so different from wild populations that the name *Salmo domesticus* has been proposed for the former (Gross, 1998). As the first steps of captivity, changed temperature during egg maturation influences egg production of their offspring (Jonsson & Jonsson, 2016), as well as changes in growth in both gonadal (Jonsson, Jonsson, & Finstad, 2014) and body sizes (Finstad & Jonsson, 2012) in later stages. Reduced genetic diversity, rapid growth, and advanced age at maturity (e.g., Fleming & Einum, 1997; Jonsson & Jonsson, 2006), as well as salmon migratory behavior (Jonsson, Jonsson, & Hansen, 1990, 2003a, 2003b), are other of the changes known to be induced by captivity in Atlantic salmon. Footprints of selection throughout the genome were already detected in early stages of domestication (after 5–6 generations of captivity; Mäkinen, Vasemägi, McGinnity, Cross, & Primmer, 2015). After 10 generations of captivity, genes involved in growth seemed to be upregulated while immune‐related and environmental information processing systems were downregulated in juveniles (Bicskei, Bron, Glover, & Taggart, 2014). Reduced responsiveness to stress was also reported after 10 hatchery generations (Solberg, Skaala, Nilsen, & Glover, 2013).

Wild anadromous Atlantic salmon populations are declining throughout all its natural distribution (Chaput, 2012). In this species, supportive breeding has resulted in introgression of hatchery/foreign genes into wild populations (Horreo et al., 2014) through the processes of inadvertent selection in the captive environment (Horreo et al., 2008), and changes induced in the population structure (Horreo, Machado‐Schiaffino, Ayllon, et al., 2011), among others. Hatchery‐reared and wild individuals differ for smolt run time (Petersson & Järvi, 2006) and straying (higher than usual values; Horreo, de la Hoz, Pola, Machado‐Schiaffino, & Garcia‐Vazquez, 2012; Jonsson et al., 2003a, 2003b; Moran, Pendas, Garcia‐Vazquez, & Izquierdo, 1994; Vasemägi et al., 2001). Regional‐scale studies in the Baltic Sea have also demonstrated that both ages at return and distribution in the Baltic main basin differ between hatchery‐reared and wild Atlantic salmon (e.g., Jutila, Jokikokko, Kallio‐Nyberg, Saloniemi, & Pasanen, 2003).

In this study, we focused on different key life‐history traits that have been documented to change significantly with domestication in Atlantic salmon: migratory behavior (determined through isotope analysis of scale tissue), straying rate (measured with genetic information), age at maturity (also determined from scales), and growth—therefore potential fitness (determined from condition factor [CF]). Stable isotope analysis offers the potential to reconstruct food webs (Hutchinson & Trueman, 2006; Syväranta, Vesala, Trask, Ruuhijärvi, & Jones, 2008), assess changes in trophic level (Wainwright, Fogarty, Greenfield, & Fry, 1993) and diet preference (Pruell, Taplin, & Cicchelli, 2003), and analyze ecosystem responses to decadal climate forcing cycles (Satterfield & Finney, 2002), to name some applications. Here, it was employed to asses changes in trophic level and to infer differences in the marine‐growing region (MacKenzie et al., 2011). Our hypothesis was these traits may be altered by captivity rearing (nurture) within a generation, before reproduction thus before genetic selection (nature). North Iberian populations were selected as a case study. In this region, supportive breeding is based on artificial spawning of wild mature individuals and rapid release of their offspring—after only 6 months in hatchery—for an average age at maturity of 3 years (Horreo et al., 2012). Therefore, most of their life they are in the wild environment.

## 2 METHODS

### 2.1 Study populations and sample collection

North Iberian Atlantic salmon populations, located at the natural southern edge of the species’ distribution, are among the most affected by environmental changes (Horreo, Machado‐Schiaffino, Griffiths, et al., 2011). Asturian salmonids are managed by the Regional Government of Asturias in collaboration with fishermen associations (especially in terms of population enhancement activities). Supportive breeding in these populations has produced up to ten million juveniles released in the rivers between 1992 and 2008 (Castillo et al., 2008). All of the breeders employed for supportive breeding in Cares and Sella rivers in 2005 were sampled (via adipose fin clips) and genotyped for analysis. In the hatcheries, located close to the rivers of origin, breeders are crossed via pooling of the ova of each female with the sperm of three males and juveniles are released 6 months later into the river from which their parents were taken. Scale samples of a portion of the returning adults (498 from River Cares and 313 from River Sella, rivers with census sizes in the year 2009 of 1,073 and 1,464 individuals, respectively) were kindly provided by sport anglers from legal salmon catches in 2007–2009. The scales were preserved dry in paper envelops. Fish length and weight were also recorded. From pedigree analysis, the early rearing of each adult (short hatchery rearing or wild) was determined (Horreo et al., 2012).

### 2.2 Age determination

The age of anadromous salmonids is given as X.Y, X, and Y being the number of years in freshwater and at sea, respectively. Y can be one (one‐sea‐winter or *grilse*, 1SW) or more (multisea‐winter, MSW) years. Adult Atlantic salmon in the study region are predominantly 1.2 plus a variable proportion of 1.1 grilse and a few 1.3, 2.1, and 2.2 individuals (Juanes, Perez, & Garcia‐Vazquez, 2007). Therefore, young of the year released in 2005 are expected to return to the river as adults in 2007, 2008, and 2009 at the ages 1.1, 1.2/2.1, and 1.3/2.2, respectively. Age reading was performed from dried scales based on scale growth circuli and double‐checked by different researchers, as published by (Horreo et al., 2012).

### 2.3 Genetic analyses

In a previous work (Horreo et al., 2012), pedigree tests were carried out with seven microsatellite loci in more than 800 salmon individuals in both rivers and the breeders employed for their production in hatcheries in order to identify hatchery descendants and their hatchery (thus river) of origin. The mentioned microsatellite information was here employed to estimate genetic variability and population differentiation between the group of individuals issued from supportive stock and the group of wild individuals. Effective number of alleles, observed and expected heterozygosity, and population differentiation (*F*_ST_ value) among groups were calculated with Genodive (Merimans & Van Tienderen, 2004). Straying rate of wild individuals between rivers was estimated from between‐river gene flow, measured as the number of migrants per generation through the mean frequency of private alleles, with the online GENEPOP software (http://genepop.curtin.edu.au).

### 2.4 Condition factor

As an indicator of growth, we have chosen CF for all the samples with available length and weight data because it is an indicator of the size and shape of fish and principally of growth rate (Gjedrem, 2005). It is also considered to indicate salmon health and fitness potential (Miller, Miller, Mills, & Sheehan, 2014). Its formula is

$$\text{CF}=[\text{weight}\times {\left({\text{length}}^{3}\right)}^{-1}]\times 100$$

### 2.5 Isotope analysis

For reasons of saving material for the collection of Atlantic salmon scales for Asturias Principality, isotope measurements were performed only for the samples with many scales, because the analytical protocol destroys the scale. A total of 63 and 62 samples from Cares and Sella rivers respectively were taken, in proportions that reflected the natural age distribution of returning adults in these populations (Juanes et al., 2007).

Acid pretreatment of scales was not performed as such pretreatment results in biologically insignificant changes in the bulk isotopic composition (Sinnatamby et al., 2009). Scales were manually cleaned using forceps to remove adherents (lipids and guanine). The last summer of growth at sea was excised as source sample to measure isotopes. In 1SW (one‐sea‐winter) fish, the summer section from the edge of the scale was sampled. In MSW (multisea‐winter) fish, the summer immediately before the final winter at sea was sampled (MacKenzie et al., 2011). Regenerated scales were not employed for analyses.

Samples were cut into small pieces and weighed to approximately 0.60 mg in 5 × 3.5 mm diameter tin cups (several scales of each individual were needed to obtain enough amount of sample material). Isotopes ratios were determined by elemental analysis using l‐glutamic acid, sugar, wheat flour, USGS40 and USGS41 as calibration standards. The resulting isotope ratios of carbon (13C/12C) and nitrogen (15N/14N) of the analyzed samples were reported versus the Pee Dee Melemnite (PDB) (carbon) and atmospheric air (nitrogen) standards [26] as:

$$\delta \left(‰\right)=103\;\left[R_{\mathrm{sample}}/R_{\mathrm{standard}}-1\right]$$

Resulting δ^13^ C values in the collagen are related with the isotopic composition of other tissues under different conditions of diet and growth rates (Satterfield & Finney, 2002; Sinnatamby et al., 2009). More importantly for this study, carbon isotopes are associated with marine feeding areas in Atlantic salmon (MacKenzie et al., 2011); differences between groups of the same age could be considered indicators of different diets. Nitrogen is more strongly fractionated than carbon during dietary assimilation (MacKenzie et al., 2011), being therefore an indicator of trophic level.

### 2.6 Statistical analysis

For determining the variables contributing more to the dataset variance, an exploratory principal component analysis (PCA) was carried out, with correlation matrix and 9,999 bootstrap. Eigenvalue cutoff was 0.7. This preliminary inspection of the dataset served to identify the main component variables and their relationships. Normality was tested in the dataset by analysis of residuals and a correlation test, and variables were transformed for normalization when required. The PCA was performed in the whole dataset, without excluding any datum.

Very divergent outliers were removed from further analysis. When it was not possible to assume normality, nonparametric tests were carried out. Comparison between multiple groups of samples was carried out using analysis of variance (ANOVA) tests or their nonparametric equivalent for medians Kruskal–Wallis. Post hoc tests were performed after significant ANOVA, as pairwise comparison between means employing *t* tests with the corresponding correction in case of unequal variances. Levene's test was applied for checking variance equality. Analyses were performed with PAST software (Hammer, Harper, & Ryan, 2001).

## 3 RESULTS

### 3.1 Genetic variation

The genetic variability of early captive and wild individuals (Table 1) was relatively high, with mean effective number of alleles (Eff_Na) of 5.105, mean observed heterozygosity (Ho) of 0.787, and mean expected heterozygosity (He) of 0.786. Assessing differences between origins and rivers (thus four groups were employed: hatchery sella, hatchery cares, wild sella, wild cares), significant differences in Eff_Na occurred between early captive and free individuals (*t* test = −4.33, *p *<* *.05), being fewer in early captive (mean Eff_Na = 4.66) than in free ones (mean Eff_Na = 5.55). Significant differences were not found between these groups either for Ho (*t* test = −0.51, *p *=* *.66) or He (*t* test = −2.68, *p *=* *.11).

**Table 1:** Genetic variability of early captive (EC) and wild (W) individuals of Sella and Cares rivers, measured as the effective number of alleles (Eff_Na), the observed heterozygosity (Ho) and expected heterozygosity (He)

Population	Eff_Na	Ho	Hs
EC‐Sella	4.741	0.794	0.781
EC‐Cares	4.578	0.768	0.763
W‐Sella	5.361	0.774	0.795
W‐Cares	5.739	0.811	0.807

*F*_ST_ values revealed significant genetic differentiation (*p *<* *.05) between early captive individuals of River Cares and all the other groups (free Cares and both free and early captive Sella individuals). No genetic differences were found between rivers even when analyzing only the wild fish (*F*_ST_ = 0.00, *p *=* *.170).

### 3.2 Principal component analysis

In the whole dataset of isotope content (carbon as δ^13^C and nitrogen as δ^15^N) and life‐history traits (age, CF, living type as early hatchery rearing in months), three principal components were significant (Eigenvalue > 0.7; Table 2). The two main components contributed a total of 69% variance (Table 2). The variables contributing more to each component were age and δ^15^N in PC1, and living type and CF in PC2. Rearing environment and CF loadings on PC2 displayed positive and negative signs and were significantly negatively correlated (*r* = −.212, *df* = 122, *p *=* *.017), and no other variable correlated significantly with the type of early living (*r* = −.083, −.023, and −.114 with age, δ^15^N, and δ^13^C respectively, all not significant). Highly significant correlations between the two isotopes and the variables age and CF were found (data are shown below).

**Table 2:** Eigenvalue and % variance contributed by each component in the principal component (PC) analysis and loadings of each variable. The loadings of the two variable with the highest contribution to each significant PC (>0.7 Eigenvalue) are in bold. Age: total age in years determined from scales; CF: condition factor; Rearing: months spent in a hatchery

	PC 1	PC 2	PC 3	PC 4	PC 5
Eigenvalue (% var)	2.42 (48.48)	1.03 (20.54)	0.83 (16.55)	0.48 (9.64)	0.24 (4.79)
Age	**0.57379**	0.13866	−0.023808	−0.32655	0.73779
CF	0.36419	−**0.32926**	**0.76361**	0.4192	−0.011167
N	**0.55643**	0.20754	0.059087	−0.4458	−0.66715
C	0.45908	0.099205	−**0.55228**	0.68268	−0.091336
Rearing	−0.13331	**0.90523**	0.32837	0.22989	0.045893

Bold values show the variables contributing more to each component

### 3.3 Migratory behavior data

Significant differences were not found between early captive and free individuals for the number of years at sea, neither among rivers (two‐way ANOVA for the factors age and living type: *F* = 0.05 and *p *=* *.83 for river, *F* = 1.00 and *p *=* *.32 for living type, no interaction between the two factors *p *=* *.99).

Because no genetic differences were found between rivers, straying rate of free individuals was here accounted through *N*_m_, that is, the number of migrants per generation, estimated from the mean frequency of private alleles present on each dataset. The result was 8.04%, clearly lower than 49% found for early captive individuals in the same populations and years (Horreo et al., 2012). For the number of individuals analyzed, the difference between these percentages was highly significant (*z* = −5.92, *p *=* *.00).

**Figure 1:**
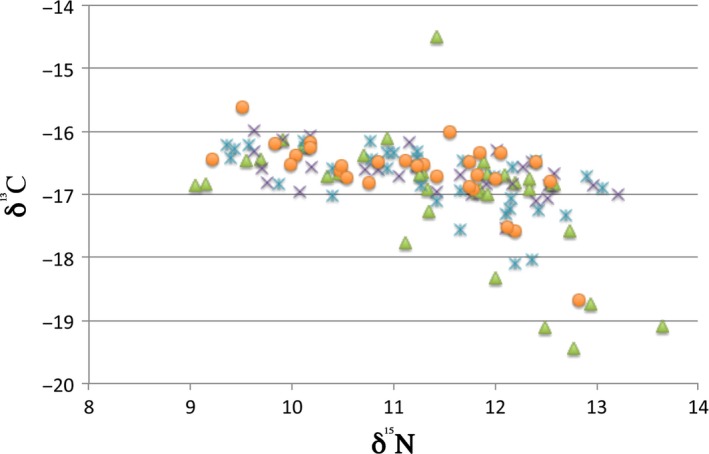
Plot of carbon (δ^13^C) on nitrogen (δ^15^N) values for wild and early captive Atlantic salmon from Sella and Cares rivers. Green: wild Sella salmon; blue: wild Cares salmon; purple: early captive in Sella; orange: early captive in Cares

Carbon (δ^13^C) and nitrogen (δ^15^N) values for wild and early captive Atlantic salmon from Sella and Cares rivers are shown in Figure 1. For the marine‐growing area in the last summer (it leaves a signature on carbon isotopes; MacKenzie et al., 2011), we tested if the free individuals from different rivers and years had significantly different mean δ^13^C values (Table 3). Two‐way ANOVA showed significant differences between rivers (*F* = 6.355, *p *=* *.014) and among age classes (*F* = 48.29, *p *≪ .001), with significant interaction (*F* = 10.31, *p *=* *.0001). Differences between ages were expected from highly significant correlation between δ^13^C and salmon age in the whole dataset (*r* = .491, *df *= 122, *p* ≪ .0001). In contrast, from two‐way ANOVA, the early captives did not differ significantly between rivers (*F* = 1.256, *p *=* *.268), but did, as expected differ significantly in early captives among ages (*F* = 19.19, *p *≪ .001), while there was no significant interaction between rivers and ages. Thus comparisons of free individuals with early captives were made independently for each year in post hoc paired tests. The wild individuals of the two rivers were pooled together when no significant differences were found between them. The difference in mean values of carbon isotopes (δ^13^C; Table 3) between wild individuals caught in 2007 in Sella and Cares rivers was significant (*t* = 4.514, *p *=* *.0009 for unequal variances), thus they were tested separately against early captives. In the River Sella, they were significantly different (*t* = 2.371, *p *=* *.037) but not in River Cares (*t* = 1.05, *p *=* *.315). For those caught in 2008, there were no significant differences between the wild of the two rivers (*t* = 1.49, *p *=* *.144) and the two rivers were joined together. The difference between the two groups (wild vs. early captive) was significant (*t* = 2.07, *p *=* *.04). Finally, for the individuals caught in 2009, there was no significant difference between Cares and Sella wild individuals (*t* = 0.581, *p *=* *.567), and the difference between wild and early captive for mean δ^13^C values was highly significant (*t* = 3.26, *p *=* *.004).

**Table 3:** Number of individuals (*n*) employed for salmon condition factor and isotope analyses depending on their river (Sella and Cares), origin (early captive: EC, wild: W), and ages (river age.sea age). Condition factor (CF), carbon (δ^13^C), and nitrogen (δ^15^N) means are given with standard deviations in brackets

	Sella					Cares			
	Age	*n*	δ^15^N	δ^13^C	CF	*n*	δ^15^N	δ^13^C	CF
Origin									
W	**1.1**	10	10.123 (0.823)	−16.318 (0.683)	0.995 (0.104)	11	10.089 (0.620)	−16.366 (0.217)	0.937 (0.127)
	**1.2**	16	11.803 (0.593)	−16.782 (0.254)	1.054 (0.064)	16	11.769 (0.578)	−16.929 (0.500)	1.065 (0.071)
	**2.2**	2	11.925 (1.152)	−17.675 (0.134)	0.953 (0.037)	6	11.785 (0.887)	−16.985 (0.605)	1.106 (0.043)
	**1.3**	5	12.770 (0.607)	−18.942 (0.426)	1.036 (0.033)	1	13.060 (0.000)	−16.900 (0.000)	1.097 (0.000)
EC	**1.1**	10	10.062 (0.435)	−16.464 (0.324)	0.949 (0.079)	10	10.168 (0.439)	−16.470 (0.223)	0.927 (0.148)
	**1.2**	16	12.011 (0.573)	−16.770 (0.330)	1.017 (0.119)	16	11.609 (0.736)	−16.607 (0.482)	0.999 (0.111)
	**2.2**	1	11.910 (0.000)	−16.830 (0.000)	1.075 (0.000)	2	11.745 (0.007)	−16.685 (0.276)	1.098 (0.116)
	**1.3**	2	12.800 (0.579)	−17.830 (1.159)	1.108 (0.082)	1	12.820 (0.000)	−18.680 (0.000)	0.977 (0.000)

### 3.4 Feeding and growth

Regarding nitrogen isotopes (Table 3), indicators of trophic level, for wild individuals, the two‐way ANOVA did not reveal differences between rivers (*F* = 1.84, *p *=* *.179), but highly significant difference among years (*F* = 54, *p *≪ 0.001), as expected from significant correlation between δ^15^N and age in the whole dataset (*r* = .755, *df *= 122, *p* ≪ .001). For early captives, however, there were significant differences in δ^15^N mean values both between rivers (*F* = 4.43, *p *=* *.04) and among years (*F* = 54.1, *p *≪ .001). In 2009, differences among the wild group and the groups of early captives entering River Sella and River Cares were not significant (*t* = 0.097, *p *=* *.923). In 2008, the differences were not significant either (*t* = 2.04, *p *=* *.05), as they were not in 2007 (*t* = 0.542, *p *=* *.616). Therefore, we compared mean values of δ^15^N between wild and early captives joining the two rivers each year. None of the comparisons were statistically significant (data not shown), thus differences in trophic level between wild and early captive individuals were not found any year.

Condition factor mean values of wild individuals (Table 3) did not differ significantly between rivers, although they did clearly among ages (Two‐way ANOVA: *F* = 0.049 with *p *=* *.826, N.S., for the factor river; *F* = 9.054, *p *=* *.0003 for the factor living type; *F* = 2.198, *p *=* *.120, N.S., for the interaction). CF correlated positively and significantly with age in the whole dataset (*r* = .402, *df *= 122, *p *=* *.003). For early captive individuals, however, no significant difference was found between ages (neither between rivers), with *F* = 0.75 and *p *=* *.39 for river effect, *F* = 3.06, *p *=* *.06 for living, not significant interaction (*F* = 0.37, *p *=* *.69). Without differences between rivers for any type of early living, the data of the two rivers were pooled together for a two‐factor ANOVA for testing early captivity and age as factors. The results showed significant differences between the two living types (*F* = 4.15, *p *=* *.04) as well as among ages, indeed (*F* = 10.45, *p *≪ .001), with no significant interaction (*F* = 0.49, *p *=* *.61). From significant negative correlation between early captivity months and CF reported above for the whole dataset, we expected lower CF for early hatchery‐reared individuals. Accordingly, individuals raised in captivity during their first months exhibited generally lower CF than wild individuals of the same age (0.942 and 0.965 for early captive and wild grilse, respectively; 1.01 and 1.06 respectively for 2‐sea‐winter individuals; the few 3‐sea‐winter individuals, however, exhibited the opposite trend with 1.046 for wild and 1.064 for early captives), although pairwise differences between living types were significant only for the most abundant 2‐sea‐age class (test for samples with unequal variance, *t* = 2.12, *p *=* *.03).

**Figure 2:**
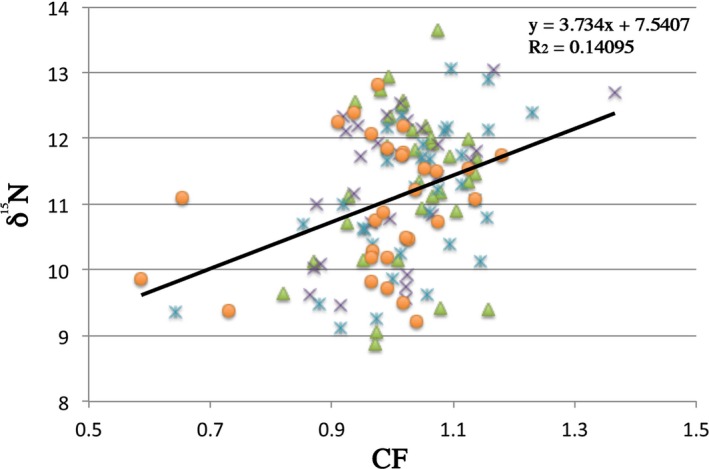
Correlation between δ^15^N and condition factor (CF) at individual level, for the region. Green: wild Sella salmon; blue: wild Cares salmon; purple: early captive in Sella; orange: early captive in Cares. δ^15^N and CF values were highly significantly correlated (*p *<* *.01)

Despite significant differences between living types (free vs. captive during the first months) for CF but not for δ^15^N, CF was highly significantly correlated with δ^15^N in this dataset (Figure 2; *p *<* *.01). This result was the same even after outliers removal (CF values lower than 0.8 and higher than 1.3; *p *<* *.01).

## 4 DISCUSSION

This work shows significant differences in both growth and migratory behavior associated with a very short period of captivity (6 months) in natural fish populations, in line with previous findings found with smolts (Jonsson et al., 2003b). The straying rate of early captive Atlantic salmon in Asturias was extremely high (49%; Horreo et al., 2012), but in comparison with it, estimated straying of wild individuals of the same populations and years was 8%. The methods employed for estimating straying were completely different because one was based on counting of strayed individuals and the other was based on the presence of allelic differences between rivers. Although there are no allele frequency differences between free‐living individuals and early captive ones, from these data it could be deduced that as a maximum only roughly 8% of the strayed individuals reproduced. An alternative (or concomitant) explanation could be that, at least in the last part of the marine migration (coastal waters nearby the natal rivers), the route may differ between early captive and free Atlantic salmon. It is known that stocked salmonids have poor homing behavior (e.g., Jonsson et al., 2003a, 2003b; Quinn, 1993); however, the enormous difference found in this study, whether due to poor reproduction of early captive or to direct differences in straying, would be attributable to only a few months of captivity. This is surprising and suggests an impact of very early domestication, even without requiring one complete generation (which is enough for genetic adaptation to captivity; Christie et al., 2011). Moreover, it implies that supportive breeding, even requiring minimal time in captivity, may change the native population structure of the species with increased gene flow between rivers. Thus, supportive breeding could not only affect the river where it is done, but also the whole region through extremely high straying rates.

Carbon isotope analysis also suggests differences between early captive and wild Atlantic salmon in the marine areas where they stayed at least the last summer before entering the river. Significant differences were found between the two life styles in 2008 and 2009, and for River Sella in 2007. MacKenzie et al. (2011) found differences between marine areas based on carbon isotopes between individuals of different ages, and here, the data also suggest the occurrence of differences between captive and wild Atlantic salmon, so relatively short exposure to the captive rearing environment appears to have the potential to affect adult behavior in the populations studied. Early nurture would therefore influence adult behavior in natural populations. The main novelty of our study is that these changes appeared within a generation, suggesting very short time of captivity produces either epigenetic changes or selection of phenotypes, which may lead to evolve differences between hatchery and wild individuals if the trait has a genetic basis, and that it is enough for initiating changes normally attributed to domestication in this species (Christie et al., 2011; Jonsson et al., 2003a, 2003b; Jutila et al., 2003; Moran et al., 1994; Vasemägi et al., 2001).

Atlantic salmon size is directly related to fitness (Garant, Dodson, & Bernatchez, 2003; Jonsson, Jonsson, & Fleming, 1996). In other studies, differences in size between captive and wild juveniles occurred after the river stage, captive‐origin Atlantic salmon released as juveniles were observed to be generally smaller than wild counterparts at the smolt life stage (De Mestral, O'Reilly, Jones, Flanagan, & Herbinger, 2013). Also reduced fitness of hatchery individuals have been found after one generation of captive breeding (Jonsson & Jonsson, 2006). In our study, we found significant differences between early captive and wild individuals for CF, which is a fish growth indicator (Gjedrem, 2005); it was significantly higher in free individuals than in the others, suggesting that wild individuals return healthier (Miller et al., 2014). Nitrogen isotopes, a signal of trophic level (Jutila et al., 2003), did not reveal differences between the two life types here compared; therefore, differences in CF would not be likely due to different feeding behavior but perhaps to some early modifications by captive rearing, as suggested by Christie et al. (2011) after one generation of domestication. Christie et al. (2016) suggested such rapid modifications could be due to adaptation to high density in hatchery conditions or, in our case, even due to differential mortality during the first months of captivity. On the other hand, nitrogen and CF were highly positive correlated (Figure 2), indeed, principally due to highly significant differences among ages for trophic level and CF occurring for this species. CF is an heritable trait that affects salmon fitness (Carlson & Seamons, 2008). Decreased CF in early captive individuals that are released in the rivers to supplement natural populations should be taken as an alert signal regarding this management practice. Especially in threatened populations such as the ones here studied (Horreo, Machado‐Schiaffino, Griffiths, et al., 2011), negative consequences such as reduced natural egg production in the artificially enhanced populations would be expected via CF decrease.

Significant reductions in the effective number of alleles were found in the individuals identified as released in the river after early captivity (Table 1), which is logical because only a fraction of the population is employed for supplementary breeding (Horreo et al., 2012). Such reduced genetic variability in supplementary stocks had been previously detected in juveniles in this region (Horreo et al., 2008) and is here reported for adults as well. Significant differences in allele frequencies (*F*_ST_) were also found between the early captive and free individuals of River Cares. If early captive individuals represented the majority of the population, these subtle differences would be highly risky. Together with altered migratory behavior, these differences are altering the population structure in the region (no genetic differences between rivers were here found, so they are being diluted via straying) and all the subjacent evolutionary processes implicated on it (Millar & Libby, 1991). Evolutionary genetic adaptation of species provides them a resistance capacity to adverse environmental conditions (Frankel & Soulé, 1981), so the observed changes would put at risk these populations, especially in adverse ecological conditions.

The results above discussed were obtained for microsatellite loci and logically are not expected to be related with natural selection due to early captivity as they correspond to two fractions of the same wild populations; it should be recalled here that the supplementary stock is produced every year from wild individuals. Nature, as represented by genetics, cannot be changed within a generation. Nurture, in this case, a short period of captivity during their early development, has notwithstanding produced significant changes in Atlantic salmon that can be seen in their adulthood, after the marine period of life. These changes may have potential implications for long‐term population stability and, for this reason, alternative methods of population enhancement such as recovering lost spawning areas (Horreo, De La Hoz, Machado‐Schiaffino, Pola, & Garcia‐Vazquez, 2011) should be explored and would be strongly recommended for conservation of the wild remnants of this valuable species.

These results may have potential implications not only regarding evolution and conservation issues of this species, but also of other species subjected to population restoration and/or enhancement processes. From our results, early stages seem to be critical for domestication‐related changes, thus early rearing in conditions as natural as possible should be seriously considered if the animals are going to be released in the wild. Endangered fish, for example, sturgeons in North America and Europe (e.g., Billard & Lecointre, 2000; Ireland, Anders, & Siple, 2002), and many amphibians (Griffiths & Pavajeau, 2008), are being reintroduced or restored based on captive‐born individuals and may benefit from taking into account the present results. Nonaquatic taxa may experience similar processes of early domestication signature as well. Reintroductions projects of carnivore mammals based on translocations of wild‐caught animals succeed more than captive‐born animals (Jule, Leaver, & Lea, 2008); among other causes, caged animals exhibit severely altered behaviors (e.g., stereotypy, Vickery & Mason, 2005; reduced behavioral flexibility, Mason et al., 2013). Despite these disturbances due to captivity, the reality is that for very endangered animals reintroduction using captive‐born individuals seems to be the only possibility of having them in the wild again. Zoo populations are recommended to maintain their evolutionary integrity through more natural mating systems that include mate choice, especially when they are employed for reintroductions in the wild (Schulte‐Hostedde & Mastromonaco, 2015). Considering the importance of early stages in domestication processes, it could be advisable as well for zoo population management to maintain individuals in wild‐like conditions since very early stages, preferably since the birth, which could increase the success of captive‐born individuals in the wild.

## AUTHOR CONTRIBUTIONS

JLH, AGV, and EGV designed and performed both data and statistical analyses. AGV, AA, and CG‐G contributed with data generation. JLH and EGV wrote the manuscript.

## CONFLICT OF INTEREST

None declared.
